# Steroid receptor coactivator 3 inhibits hepatitis B virus gene expression through activating Akt signaling to prevent HNF4α nuclear translocation

**DOI:** 10.1186/s13578-019-0328-5

**Published:** 2019-08-13

**Authors:** Ming Li, Yi Wang, Xiaochun Xia, Pingli Mo, Jianming Xu, Chundong Yu, Wengang Li

**Affiliations:** 1 Department of Hepatobiliary and Pancreatic & Organ Transplantation Surgery, Xiang’an Hospital of Xiamen University, School of Medicine, Xiamen University, Xiamen, 361012 Fujian China; 2 State Key Laboratory of Cellular Stress Biology, School of Life Sciences, Xiamen University, Xiamen, 361012 Fujian China; 3 Department of Medical Technology, Xiamen Medical College, Xiamen, China; 4 Department of Molecular and Cellular Biology, Baylor College of Medicine, Houston, TX USA

**Keywords:** SRC-3, Hepatitis B virus, Akt, HNF4α

## Abstract

**Background:**

Chronic hepatitis B virus (HBV) infection is one of the most serious global public health problems. The role of steroid receptor coactivator 3 (SRC-3) in HBV biosynthesis is unknown. The aim of this study is to investigate the function of SRC-3 in regulating HBV biosynthesis both in vitro and in vivo and to identify the underlying mechanism.

**Results:**

In this study, we found that knockdown of SRC-3 could increase the levels of HBV mRNA and HBV proteins HBsAg and HBeAg in human liver cancer cell line HepG2 transfected with pHBV1.3 plasmids. In contrast, enforced expression of SRC-3 in SRC-3-knockdown HepG2 cells reduced the levels of HBV mRNA and HBV proteins HBsAg and HBeAg. Knockdown of SRC-3 dampened the Akt signaling, which has been shown to play a negative role in HBV transcription. Ectopic expression of constitutively activated Akt impaired the enhancement of HBV transcription by SRC-3 knockdown, indicating that SRC-3 inhibits HBV transcription by enhancing Akt signaling. Both SRC-3 and constitutively activated Akt could inhibit hepatocyte nuclear factor 4α (HNF4α)-mediated upregulation of HBV core promoter activity by preventing HNF4α nuclear translocation. Consistent with the in vitro results, in an in vivo chronic HBV replication mouse model developed by hydrodynamic injection of pHBV1.3 plasmids into mouse tail vein, enforced expression of SRC-3 in mouse liver reduced the levels of HBV mRNA in the liver and HBV antigens in serum, whereas knockout of SRC-3 in mouse increased the levels of HBV mRNA in the liver and HBV antigens in the serum.

**Conclusion:**

Our study suggests that SRC-3 inhibits HBV gene expression by activating Akt signaling to prevent HNF4α nuclear translocation.

**Electronic supplementary material:**

The online version of this article (10.1186/s13578-019-0328-5) contains supplementary material, which is available to authorized users.

## Introduction

Hepatitis B virus (HBV) infection is the most common chronic viral infection in the world. Despite significant improvement in the management of HBV, it is still a global public health problem [1]. About 2 billion people were infected with HBV worldwide and more than 350 millions have become chronic infection [2, 3]. Chronic HBV infection greatly increases the risk of chronic liver disease, including hepatitis, fibrosis, cirrhosis, and liver cancer [3].

Hepatitis B virus is a small enveloped hepatotropic DNA virus that belongs to the *Hepadnaviridae* family and its life cycle involves both DNA and RNA intermediates [3]. HBV genome (3.2 kb) is circular partial duplex DNA, also calling relaxed circular (rc) DNA. After HBV entering hepatocyte, rcDNA genome is released into the nucleus. In hepatocyte nucleus, cellular enzymes convert the rcDNA to a covalently closed circular DNA (ccc DNA) [4, 5]. cccDNA is a key component in the HBV life cycle, since it is the template for pregenomic/precore RNAs (3.5-kb) and subgenomic RNAs (2.4, 2.1, 0.7-kb) [4, 5]. HBV genome cccDNA has widely overlapping open reading frames (ORFs) and regulatory elements overlap with coding sequence, therefore a 3.2 kb HBV genome has 5 transcriptions depending on different promoter regions (both 3.5, 2.4, 2.1 and 0.7-kb) and some transcriptions have multiple ORFs depending on different translation regions [5]. The pregenomic RNA (pgRNA) serves as mRNAs for generating hepatitis B core antigen (HBcAg) and polymerase/reverse transcriptase protein and is the template of reverse transcription to produce the HBV DNA [5]. HBcAg protein constitutes viral capside and viral DNA genome and virally encoded polymerase were inside of the capside [5]. The Precore RNA (preRNA) serves as an mRNA for generating the secreted hepatitis B e antigen (HBeAg) and the function of HBeAg may be involved in immune toleration [5]. The 2.4 kb subgenomic RNA serves as an mRNA for generating large HBsAg (hepatitis B surface antigen) protein and 2.1 kb subgenomic RNA serves as an mRNA for generating middle and small HBsAg [5]. The viral capside is surrounded by a lipid envelope containing HBsAg. HBsAg and HBeAg are viral markers detected in serum [5]. The 0.7-kb subgenomic RNA serves as an mRNA for generating HBx protein which contributes to HBV infection and oncogenic potential.

HBV replicates by reverse transcription of 3.5-kb pgRNA, therefore the level of this transcript is a primary determinant of viral biosynthesis [4]. Extensive studies demonstrate that a number of ubiquitous transcription factors and liver-enriched transcription factors/nuclear receptors bind to the HBV promoter/enhancer regions and regulate the activity of these regulator elements, and in turn control the transcription of HBV gene [4]. Hepatocyte nuclear factor 4α (HNF4α) is a member of the nuclear receptor family and regulates the expression of 44% of hepatocyte-specific genes [6]. Overexpression of HNF4α increases the levels of HBV mRNA in hepatoma cell lines and non-hepatic cell lines [7–9]. HNF4α increases the synthesis of HBV pregenomic RNA by activating HBV promoter in hepatoma cell huh-7 [10]. Conditional depletion of HNF4α in the liver decreases the HBV transcription and replication in HBV transgenic mouse model of chronic infection [11]. These results suggest that HNF4α is a major regulator of pregenomic RNA transcription and HBV replication, consequently determining the viral biosynthesis. The phosphatidylinositol 3-kinase (PI3K)-protein kinase B/Akt signaling pathway plays crucial role in cell proliferation, differentiation, and survival [12]. Activation of Akt inhibits HBV transcription and replication [13, 14]. Akt acts at HNF4α to decrease HBV transcription [14].

Steroid receptor coactivator 3 (SRC-3/ACTR/AIB-1/pCIP/TRAM-1), is a member of p160 coactivator family. SRC-3 not only interacts with nuclear hormone receptors but also interacts with other transcription factors to enhance their downstream target gene transcription [15]. SRC-3 is overexpressed in human hepatocellular carcinoma (HCC) and promotes tumor progression by enhancing Akt signaling [15, 16]. Our previous study has demonstrated that HBx protein, a regulator of HBV replication, stabilizes SRC-3 protein and cooperates with it to promote human HCC cell invasiveness [17], indicating that there exist crosstalk between SRC-3 and HBV in the liver. In this study, we investigate the role of SRC-3 in regulating HBV biosynthesis in vitro and in vivo and identify the underlying mechanism.

## Materials and methods

### Animals and cell cultures

WT and SRC-3^−/−^ male mice (6–8 weeks old) were used for hydrodynamic injection experiments. All animal protocols were approved by Animal Care and Use Committee of Xiamen University. HepG2 cells were cultured in DMEM medium supplemented with 10% FBS.

### Plasmids and transfection

pGL3-EnII/Cp-luc plasmid was constructed in our laboratory. HBV Enhancer II/core promoter element (1399–1890 nt) was cloned into pGL3-basic vector expressing firefly luciferase to generate pGL3-EnII/Cp-luc. Constitutively active Akt expression plasmid (Akt1-T308D/S473D) was provided by Dr. Jianming Xu (Baylor College of Medicine, USA). Transfection was performed at the cell-density of 60–80% per well using Lipofectamine 2000 according to the manufacturer’s instruction (Invitrogen).

### Measuring of the levels HBsAg and HBeAg proteins

The protein levels of HBsAg and HBeAg in culture media or animal serum were measured by ELISA kit for HBsAg (Cat No. 05.02.01.012; InTec Products, China) and HBeAg (Cat No. 05.02.03.001; InTec Products, China), respectively. Culture media were collected for centrifugation at 13,000×*g* for 10 min to remove cell debris. The blood freshly collected was placed at room temperature for 1 h to clot, followed by centrifugation at 5500×*g* for 5 min to harvest the serum.

### Western blot analysis

Western blotting analysis was performed as previously described [16]. The antibody against SRC-3 (Cat No. 2126S; CST, USA), p-AKT (Cat No. 4060S; CST, USA), AKT (Cat No. 9272S; CST, USA), Tublin (Cat No. 2148S; CST, USA), GFP (Cat No. 2956T; CST, USA) and PARP (Cat No. 9532T; CST, USA) were purchase from Cell Signal Technology. The antibody against human HNF4α (Cat No. ab92378; Abcam, UK) and mouse HNF4α (Cat No. ab41898; Abcam, UK) was purchase from abcam. The β-actin antibody (Cat No. A5441; Sigma Aldrich, USA) was purchase from Sigma Aldrich.

### Quantitative real-time PCR (qPCR)

Total RNA was isolated with Trizol reagent (Invitrogen) according to the manufacturer’s instructions. The cDNA was synthesized from 2 μg of total RNA using MMLV transcriptase (ToYoBo, Shanghai, China) with random primers. Real-time PCRs were performed using SYBR Premix ExTaq (TaKaRa, Dalian, China). Specific primers used to detect HBV total mRNAs by real-time PCR were as follows: forward primer, 5′-ACGTCCTTTGTTTACGTCCCGT-3′, reverse primer, 5′-CCCAACTCCTCCCAGTCCTTAA-3′ [18]. All the other real-time primers are listed as follows: IGF1 forward primer, 5′-TTTATTTCAACAAGCCCACA-3′, IGF1 reverse primer, 5′-TCTCCAGCCTCCTTAGATC-3′; IRS1 forward primer, 5′-CTTCGGTGTCTGGTTCCC-3′, IRS1 reverse primer, 5′-ATAGTTGCTTAGCTCCTCCTCA-3′; IRS2 forward primer, 5′-GGCTTGGTCGGTTGTCCTGG-3′, IRS2 reverse primer, 5′-CCTCACGTCGATGGCGATGTAG-3′.

### Luciferase reporter assay

pGL3-EnII/Cp-luc plasmid was tranfected into HepG2 cells together with pCR3.1-Rluc. Cells were assayed at 48 h post-transfection for luciferase activity and normalized to Renilla luciferase activity using a dual luciferase reporter assay system (Promega, Madison, WI, USA).

### Hydrodynamic injection (HI) mouse model

To test HBV replication in vivo, mice were subjected to hydrodynamic injection with 5 mice per group. The HI method has been described previously [19]. Briefly, WT and SRC-3^−/−^ male mice were injected with 10 μg pHBV1.3 plasmid together with 1 μg pPCR3.1-Renilla-luciferase plasmid DNA in PBS with a volume of 8% (w/v) bodyweight via tail vein within 5–8 s. WT male mice were injected with 10 μg pHBV1.3 plasmid and 1 μg PCR3.1-Renilla-luciferase plasmid together with 10 μg pPCR3.1-SRC-3 plasmid or the same molar control plasmid DNA in PBS with a volume of 8% (w/v) body weight via tail vein within 5–8 s. The efficiency of transfection was normalized to the activity of Renilla-luciferase. To assess the transfection efficiency of HI mouse model in this study, 10 μg PCR3.1-GFP plasmids were injected into mouse and liver cells expressing GFP gene were identified by immunochemistry. There were approximately 25% of cells expressing GFP gene in mouse liver section (Additional file 1: Figure S1).

### Statistical analysis

Statistical analysis was performed using SPSS 17.0 for Windows. Differences between groups were evaluated with t-test. p value < 0.05 was considered significant.

## Results

### SRC-3 inhibits HBV biosynthesis in HepG2 cells

**Fig. 1:**
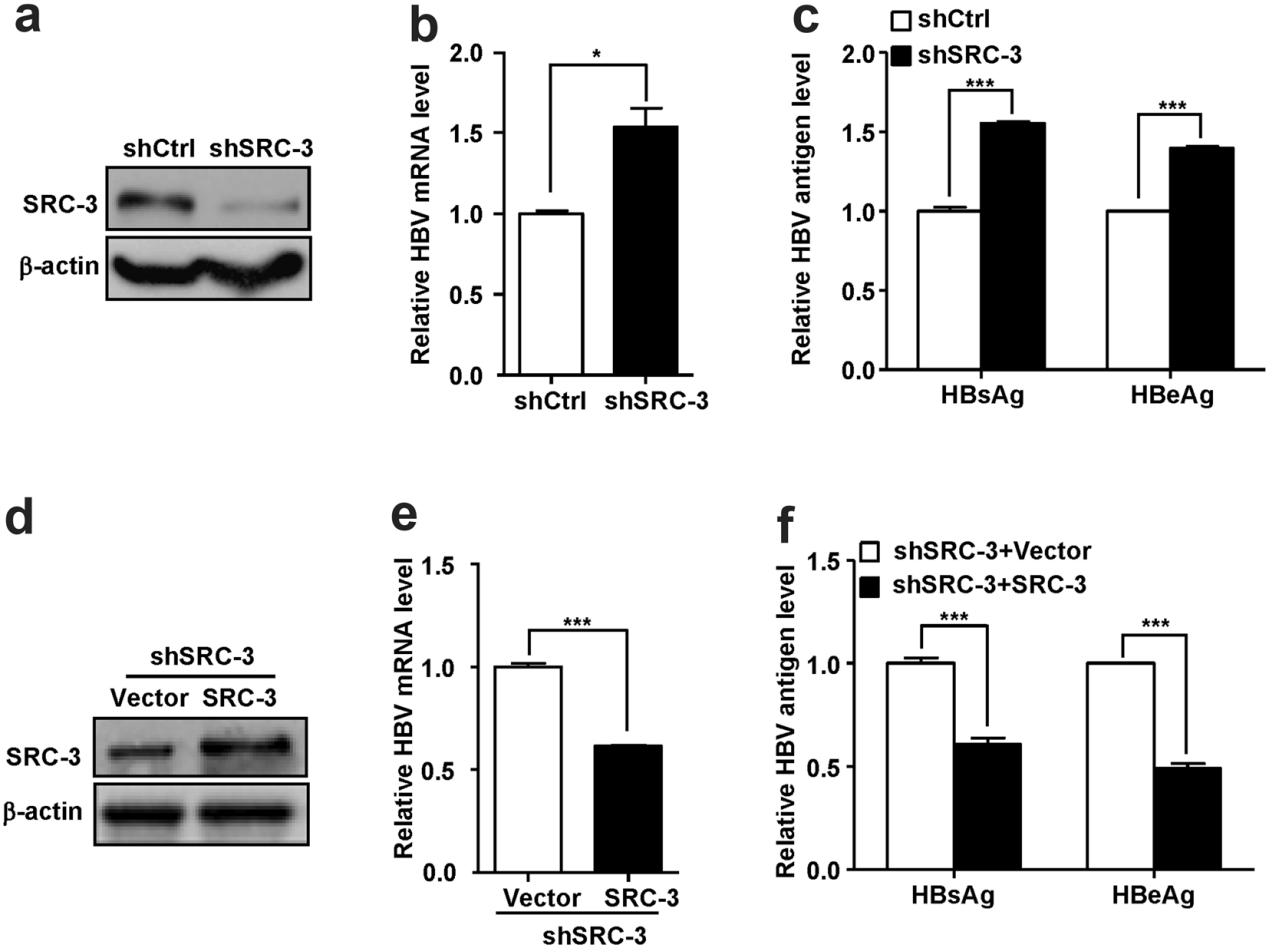
SRC-3 inhibits HBV biosynthesis in HepG2 cells. **a**–**c** Knockdown of SRC-3 increased the mRNA levels of HBV and the protein levels of HBsAg and HBeAg. Stable SRC-3-knockdown and control HepG2 cells were transfected with pHBV1.3 plasmids, respectively. The SRC-3 protein was tested by western blot (**a**), the mRNA level of HBV was quantified by real-time PCR (**b**), and the protein of HBsAg and HBeAg was measured by ELISA (**c**). **d**–**f** Ectopic expression of SRC-3 in SRC-3-knockdown HepG2 cells reduced the mRNA levels of HBV and the protein levels of HBsAg and HBeAg. Stable SRC-3-knockdown HepG2 cells were transfected with SRC-3/pHBV1.3 plasmids or control/pHBV1.3 plasmids for 48 h. The protein levels of SRC-3 were detected by western blot (**d**), the mRNA level of HBV was quantified by real-time PCR (**e**), and the protein of HBsAg and HBeAg was measured by ELISA (**f**). All experiments were repeated at least 3 times independently. *p < 0.05, ***p < 0.001

To investigate the role of SRC-3 in HBV biosynthesis, we established stable SRC-3-knockdown HepG2 cells using pSuper plasmid containing shRNA against SRC-3 or scramble control shRNA as described previously [16]. The shSRC-3, but not scramble shRNA (shCtrl) efficiently knocked down the expression of SRC-3 in HepG2 cells (Fig. 1a). The HBV biosynthesis model was established by transfecting pHBV1.3 plasmids into control and SRC-3-knockdown HepG2 cells, respectively. Downregulation of SRC-3 increased HBV mRNA levels in cells (Fig. 1b), as well as the protein levels of HBsAg and HBeAg in culture media (Fig. 1c), indicating that SRC-3 could negatively regulate HBV transcription and viral protein production. Furthermore, we performed SRC-3 rescue experiment to confirm the role of SRC-3 in HBV biosynthesis (Fig. 1d). Stable SRC-3-knockdown cells were transiently transfected with SRC-3 expression plasmids or control plasmids together with pHBV1.3 plasmids for 48 h, the protein levels of SRC-3 were detected by Western blot, the mRNA levels of HBV were quantified by real-time PCR, and the protein levels of HBsAg and HBeAg were measured by ELISA. As expected, the levels of HBV mRNA as well as HBsAg and HBeAg proteins were downregulated when overexpressing SRC-3 in SRC-3-knockdown cells (Fig. 1e, f). These results suggest that SRC-3 inhibits HBV gene transcription and viral protein production, consequently reducing HBV biosynthesis in HepG2 cells.

### SRC-3 activates Akt to inhibit HBV biosynthesis

**Fig. 2:**
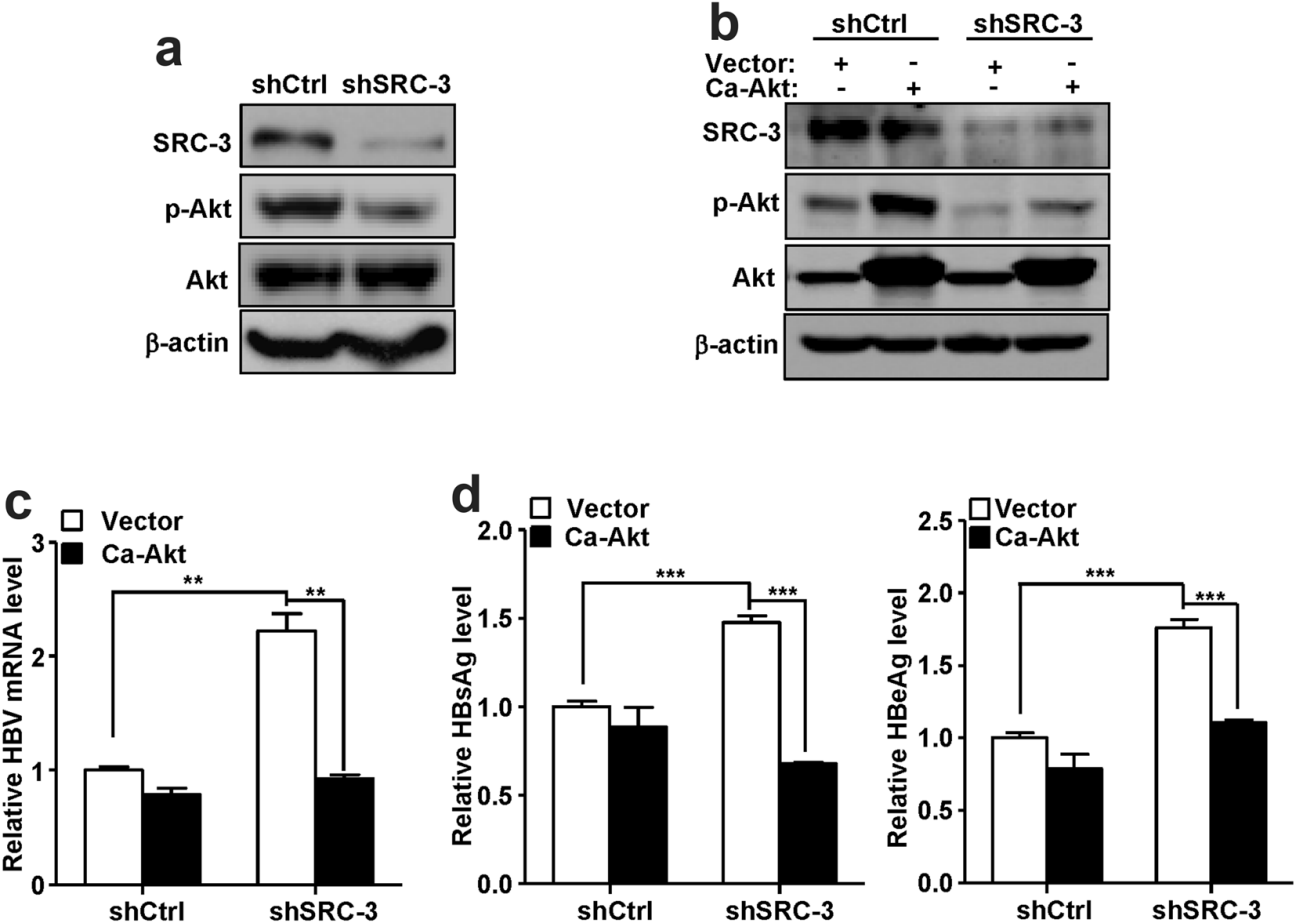
SRC-3 activates Akt signaling to inhibit HBV gene expression. **a** Knockdown of SRC-3 decreased phosph-Akt expression in HepG2 cells. The expression of p-Akt and t-Akt were analyzed by western bolt. β-actin was served as a loading control. **b**–**d** Ectopic expression of constitutively active Akt (CA-Akt) reduced the mRNA levels of HBV and the protein levels of HBsAg and HBeAg. SRC-3-knockdown or control HepG2 cells were transfected with CA-Akt and pHBV1.3 plasmids for 48 h, and then the protein levels of SRC-3, p-Akt and t-Akt were detected by western blot (**b**), the mRNA level of HBV was quantified by real-time PCR (**e**), and the protein levels of HBsAg and HBeAg was measured by ELISA (**d**). All experiments were repeated at least 3 times independently. **p < 0.01, ***p < 0.001

It has been demonstrated that activation of Akt inhibits HBV gene transcription and replication [13, 14]. Previous study has demonstrated that SRC-3 up-regulates the transcription of multiple genes in insulin-like growth factor (IGF)/AKT signaling pathway [such as IGF1, insulin receptor substrate (IRS) 1 and IRS2] to activate AKT signaling [20]. Consistent with previous studies [16, 20], we found that downregulation of SRC-3 significantly reduced IGF1, IRS1, and IRS2 mRNA expression (Additional file 2: Figure S2) and the level of phosphorylated Akt (activated form of Akt) was significantly decreased in SRC-3-knockdown cells (Fig. 2a), implicating that SRC-3 may inhibit HBV biosynthesis by activating Akt. To test this hypothesis, we rescued Akt activity in SRC-3-knockdown cells by transfecting constitutively active Akt (Ca-Akt) expression plasmids (Fig. 2b), and then examined HBV biosynthesis. The results showed that the levels of HBV mRNA (Fig. 2c) and HBsAg and HBeAg proteins (Fig. 2d) were significantly decreased after transfection of Ca-Akt expression plasmids into SRC-3-knockdown cells. Collectively, these results suggest that SRC-3 inhibits HBV biosynthesis by enhancing Akt signaling.

### SRC-3 inhibits HBV promoter activity through regulating Akt/HNF4α signal axis

**Fig. 3:**
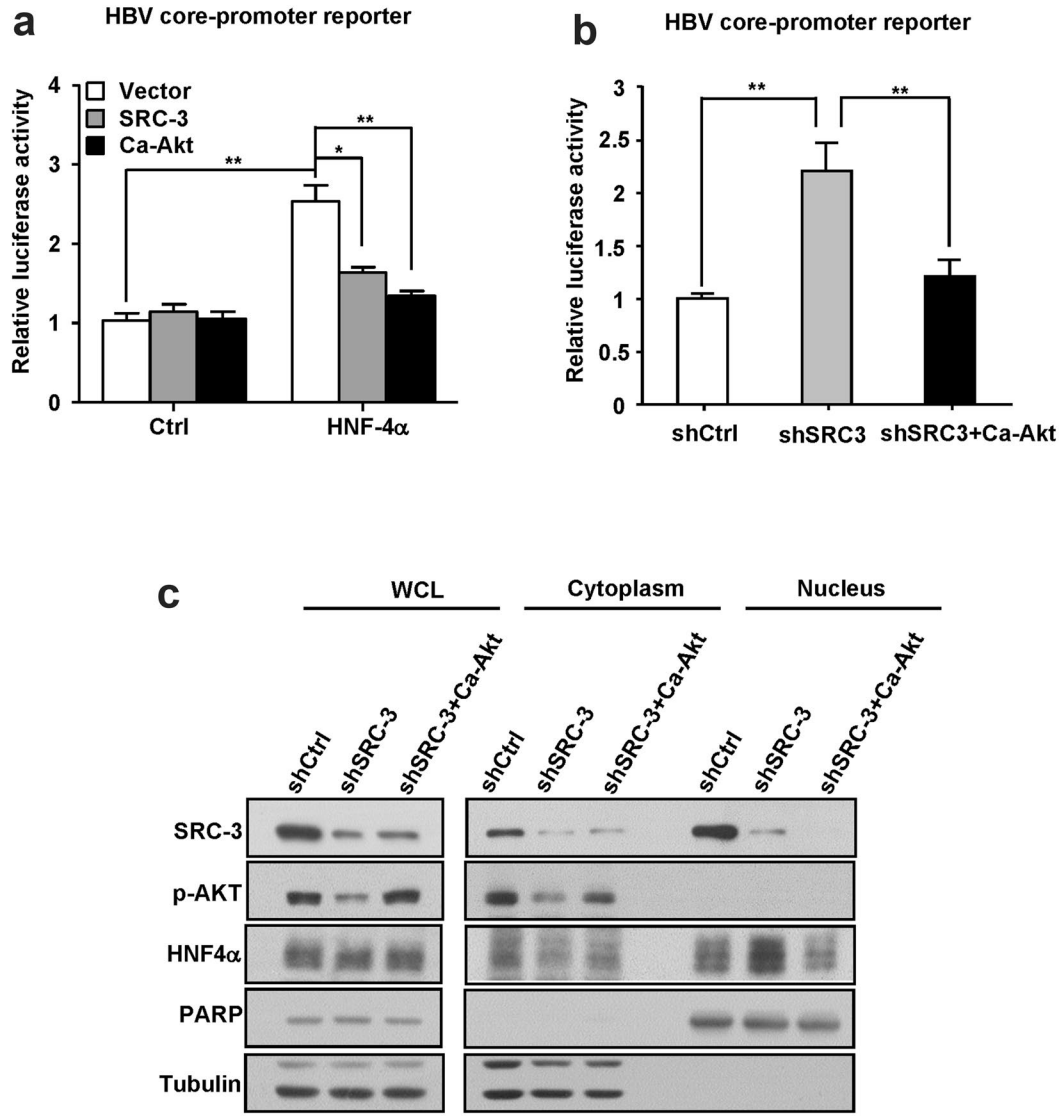
SRC-3 inhibits HNF4α-induced HBV core-promoter activity. **a** Ectopic expression of SRC-3 or CA-Akt inhibited HNF4α-induced HBV core-promoter activity in HepG2 cells. **b** Transfection of CA-Akt into SRC-3-knockdown cells inhibited HBV core promoter activity. **c** Transfection of CA-Akt into SRC-3-knockdown cells prevented the nuclear translocation of HNF4α. Control constructs were transfected into control cell or SRC-3-knockdown cells and CA-Akt were transfected into SRC-3-knockdown cells for 48 h, then cell fractionation was carried out and fractions were analyzed by western blot. All experiments were repeated at least 3 times independently. *p < 0.05, **p < 0.01

It has been reported that hepatocyte-specific transcription factor HNF4α increases the synthesis of HBV pregenomic RNA by activating HBV core promoter [7, 10], but the positive effect of HNF4α on HBV RNA synthesis could be attenuated by Akt signaling [14]. To determine whether SRC-3 could inhibit HNF4α-induced HBV biosynthesis, we cloned HBV Enhancer II/core promoter element (1399–1890 nt) into pGL3-basic firefly luciferase reporter plasmids to generate pGL3-EnII/Cp-luc, and then transfected pGL3-EnII/Cp-luc plasmids along with control plasmids, SRC-3 expression plasmids, or constitutively active Akt (Ca-Akt) expression plasmids into HepG2 cells, respectively. As shown in Fig. 3a, HNF4α enhanced the HBV enhancer II/core promoter activity as expected, but overexpression of SRC-3 or Ca-Akt inhibited HNF4α-induced HBV-core promoter activity. In contrast, knockdown of SRC-3, which reduced Akt activity (Fig. 3c), increased HBV-core promoter activity (Fig. 3b). Restoration of Akt activity in SRC-3-knockdown cells by transfecting Ca-Akt expression plasmids repressed HBV-core promoter activity (Fig. 3b). These results suggest that SRC-3 inhibits HNF4α-induced HBV-core promoter activity by activating Akt signaling. Knockdown of SRC-3 had no effect on HNF4α expression, but promoted the nuclear translocation of HNF4α (Fig. 3c). Restoration of Akt activity in SRC-3-knockdown cells inhibited the nuclear translocation of HNF4α (Fig. 3c). These results suggest that SRC-3 inhibits HBV promoter activity by preventing HNF4α nuclear translocation via activation of Akt signaling.

### SRC-3 inhibits HBV biosynthesis in vivo

**Fig. 4:**
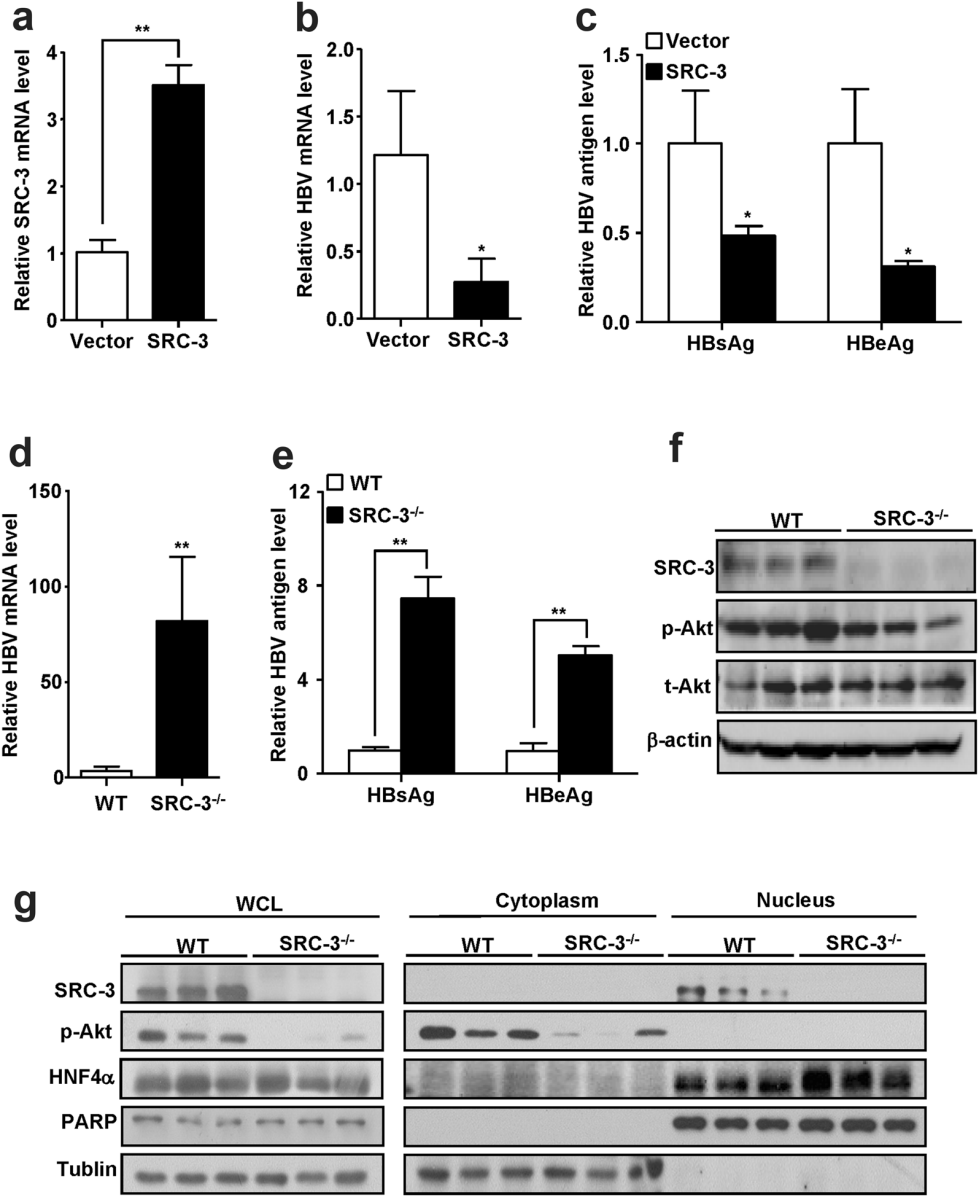
SRC-3 inhibits HBV gene expression in vivo in a hydrodynamic injection (HI) mouse model. **a**–**c** Ectopic expression of SRC-3 in mouse liver reduced the mRNA levels of HBV in the liver and the protein levels of HBsAg and HBeAg in the serum. The control plasmids and SRC-3 expression plasmids, together with pHBV1.3, were transduced into mice by tail vein hydrodynamic injection, respectively. The transfection efficiency was normalized to Renilla luciferase. The mRNA levels of SRC-3 (**a**) and HBV (**b**) were quantified by real-time PCR. The serum concentrations of HBsAg and HBeAg proteins were measured by ELISA (**c**). **d**, **e** Knockout of SRC-3 in mouse increased the mRNA levels of HBV in the liver (**d**) and the protein levels of HBsAg and HBeAg in the serum (**e**). Wild-type (WT) and SRC-3^−/−^ mice were injected with pHBV1.3 plasmids by tail vein hydrodynamic injection, respectively. The serum concentrations of HBsAg and HBeAg were measured by ELISA; and the mRNA level of HBV was quantified by real-time PCR. **f** Knockout of SRC-3 in mouse reduced the levels of p-Akt in the liver. The protein levels of SRC-3, p-Akt, and t-Akt in WT and SRC-3^−/−^ liver tissues were detected by western bolt. **g** SRC-3 deficiency promoted the nuclear translocation of HNF4α. Mouse liver fractionation was carried out and fractions were analyzed by western blot. All experiments were repeated twice independently. *p < 0.05, **p < 0.01

To detect the effect of SRC-3 on HBV biosynthesis in vivo, hydrodynamic injection (HI) mouse model was adopted to co-express HBV and SRC-3 in the liver. The mRNA levels of SRC-3 in the liver were significantly upregulated after intravenous injection of SRC-3 expression plasmids (Fig. 4a), whereas the HBV mRNA levels in the liver and the protein levels of HBsAg and HBeAg in the serum were decreased (Fig. 4b, c). Furthermore, SRC-3^−/−^ mice were used to determine the effect of SRC-3 deficiency on HBV biosynthesis by intravenously injecting pHBV1.3 plasmids. SRC-3 deficiency increased the mRNA levels of HBV in the liver (Fig. 4d), as well as the proteins levels of HBsAg and HBeAg in the serum (Fig. 4e). These results suggest that SRC-3 indeed can inhibit HBV transcription and viral protein production in vivo. In consistent with in vitro data, SRC-3 deficiency decreased the levels of phosphorylated Akt in the liver (Fig. 4f), but promoted the nuclear translocation of HNF4α (Fig. 4g), indicating that SRC-3 inhibited HBV biosynthesis in vivo by activating Akt signaling to prevent HNF4α nuclear translocation.

## Discussion

Our previous study showed that SRC-3 was overexpressed in human HCC specimens and promoted HCC progression, and HBV X protein (HBx), a regulator of HBV replication, interacted and stabilized SRC-3 protein [16, 17]. However, the role of SRC-3 in HBV biosynthesis remains unclear. In this study, our results showed that knockdown of SRC-3 increased the expression of HBsAg and HBeAg proteins in cell media and HBV mRNA in cells; consistently, knockout of SRC-3 in mouse increased the expression of HBsAg and HBeAg proteins in the serum and HBV mRNA in the liver. In contrast, overexpression of SRC-3 decreased the expression of HBsAg, HBeAg proteins and HBV mRNA in vitro and in vivo. Our study demonstrates that SRC-3 inhibits HBV gene transcription and viral protein production.

The level of HBV gene transcription determines the HBV gene expression and HBV biosynthesis [4]. Activation of PI3 K/Akt frequently happens in numerous types of human cancers and promotes tumor progression [21, 22]. It has been shown that activation of Akt is one of the most consistent characteristic of HBV-induced HCC in a colligated microarray assay [23]. However, activation of Akt inhibits HBV gene transcription and consequently decreases HBV biosynthesis [13, 14]. Our results showed that knockdown of SRC-3 reduced phosphorylated-Akt expression, but increased HBV gene transcription and protein expression; restoration of Akt activity in SRC-3-knockdown cells repressed HBV gene transcription and protein expression. These results indicate that SRC-3-mediated decrease of HBV biosynthesis is linked to the increase of Akt activity.

A number of liver-enriched transcription factors/nuclear receptors determines HBV gene transcription level and represents a crucial determinant of HBV liver tropism [4]. It has been shown that HNF4α promotes HBV gene transcription and consequently increases HBV replication [7–10]. In addition, activation of Akt can phosphorylate HNF4α and result in the translocation of HNF4α out of the nucleus [24]. Therefore, HNF4α acts at downstream of Akt signaling for regulation of HBV transcription [14]. Our results showed that both SRC-3 and Akt inhibited HNF4α-induced HBV-enhancer II/core promoter activity; restoration of AKT activity in SRC-3-knockdown cells reduced the nuclear translocation of HNF4α protein as well as HBV-core promoter activity (Fig. 3b, c). These results indicate that SRC-3 inhibits HBV gene transcription at least in part through activating Akt to inhibit HNF4α nuclear translocation.

**Fig. 5:**
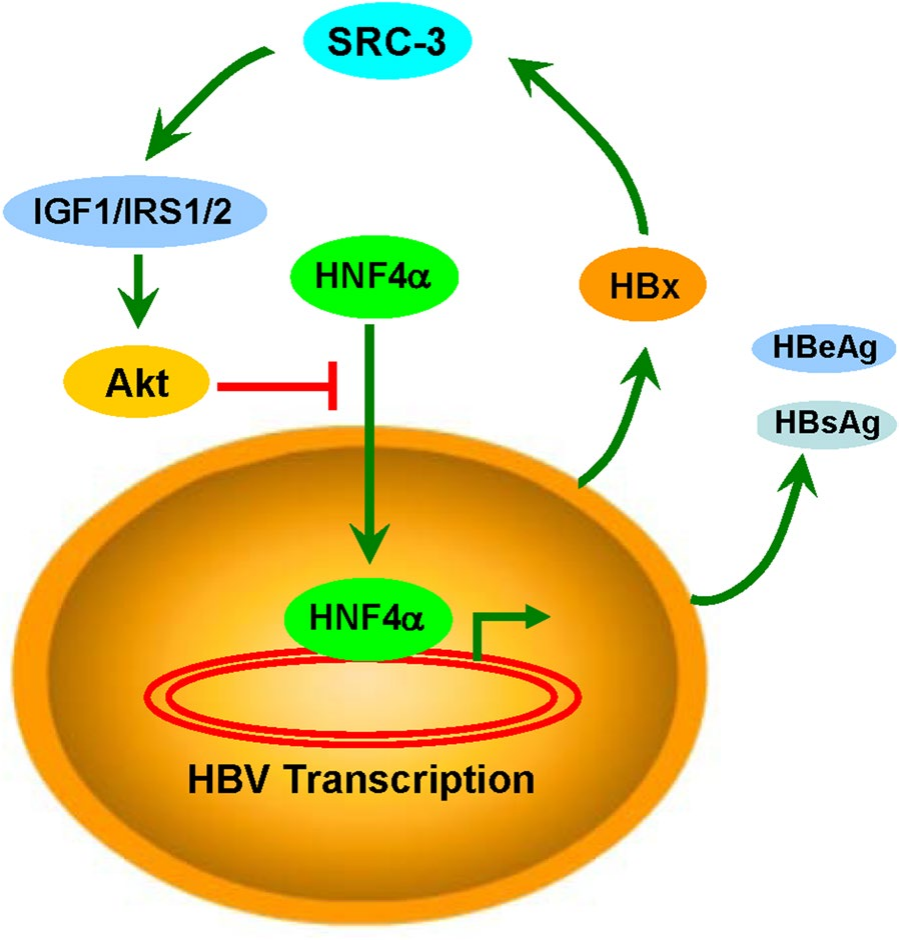
A schematic diagram depicting the molecular mechanism by which SRC-3 inhibits HBV gene expression. SRC-3 inhibits HBV gene expression by activation of Akt signaling via inducing the expression of IGF1, IRS1, and IRS2 to prevent HNF4α nuclear translocation; HBV gene expression results in HBx protein production to stabilize SRC-3 protein, leading to a feedback inhibition of HBV gene expression

We previously reported that HBx could interact with SRC-3 protein to prevent SRC-3 protein degradation [16, 17]. Based on our previous and current studies, we propose a working model to depict the molecular mechanism by which SRC-3 inhibits HBV gene expression: SRC-3 inhibits HBV gene expression by activation of Akt signaling via inducing the expression of IGF1, IRS1, and IRS2 to prevent HNF4α nuclear translocation; HBV gene expression results in HBx protein production to stabilize SRC-3 protein, leading to a feedback inhibition of HBV gene expression (Fig. 5). Taken together, our study demonstrates an important role of SRC-3 in the control of HBV.

## Additional files

**Additional file 1: Figure S1.** Histochemical analysis of GFP gene expression in mouse liver. 10 μg pPCR3. 1-GFP plasmids were injected into mouse and the animal was killed 8 h after injection. Mouse liver cells expressing GFP gene were identified by immunochemistry.

**Additional file 2: Figure S2.** Downregulation of SRC-3 significantly inhibits the expression of IGF1, IRS1, and IRS2. The mRNA levels of IGF1, IRS1, and IRS2 were quantified by real-time PCR in stable SRC-3-knockdown and control HepG2 cells, respectively. *p < 0.05, **p < 0.01, ***p < 0.001.

## Data Availability

The datasets supporting the conclusions of this article are included within the article.
