# Supplementary figures and images for: Steroid receptor coactivator 3 inhibits hepatitis B virus gene expression through activating Akt signaling to prevent HNF4α nuclear translocation
Source: Cell Biosci. 2019 Aug 13;9:64. doi: 10.1186/s13578-019-0328-5 (PMC6692928; doi:10.1186/s13578-019-0328-5)

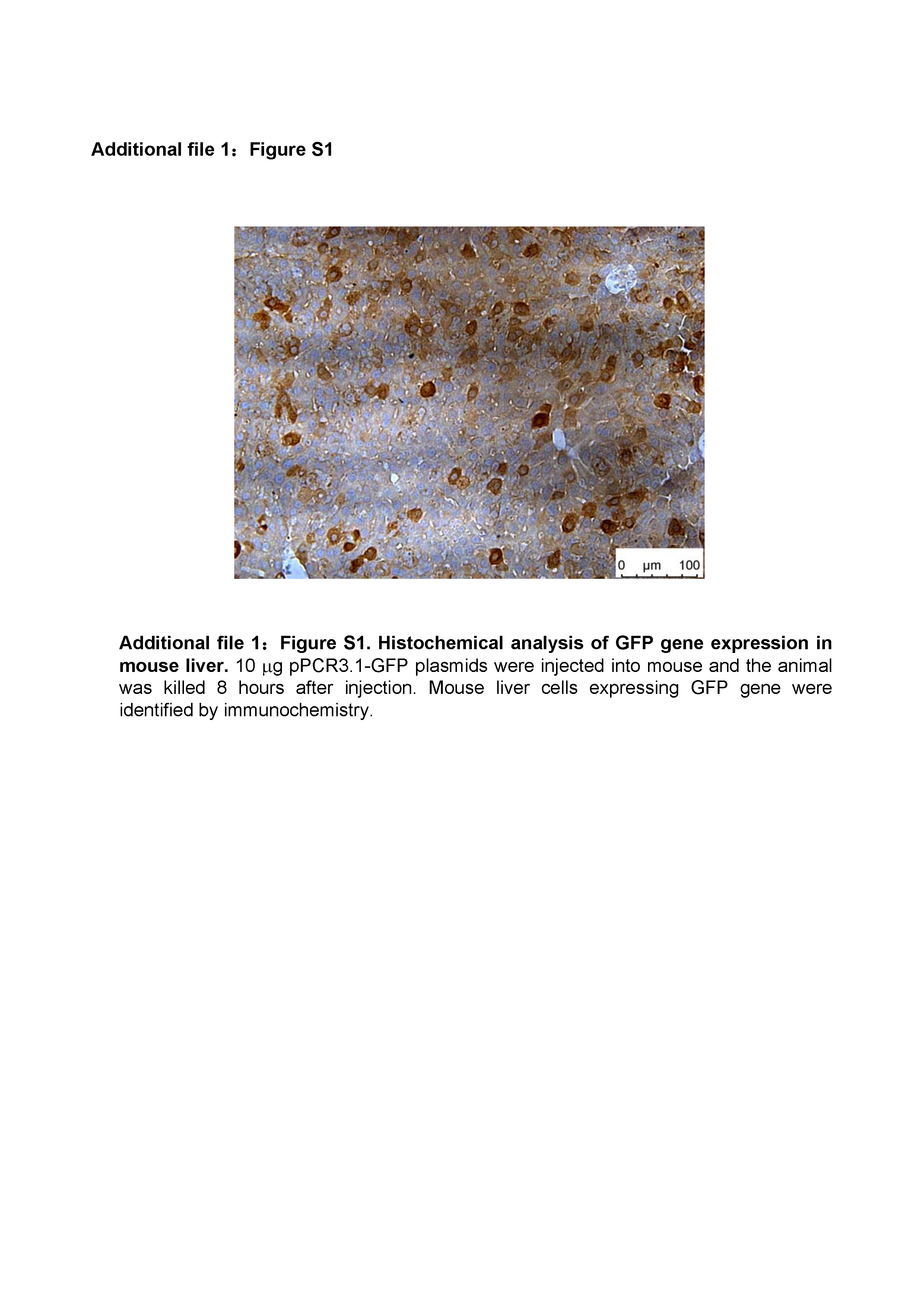

Supplement: Supplementary file 1 — Additional file 1: Figure S1. Histochemical analysis of GFP gene expression in mouse liver. 10 μg pPCR3. 1-GFP plasmids were injected into mouse and the animal was killed 8 h after injection. Mouse liver cells expressing GFP gene were identified by immunochemistry. [file 13578_2019_328_MOESM1_ESM.tiff]

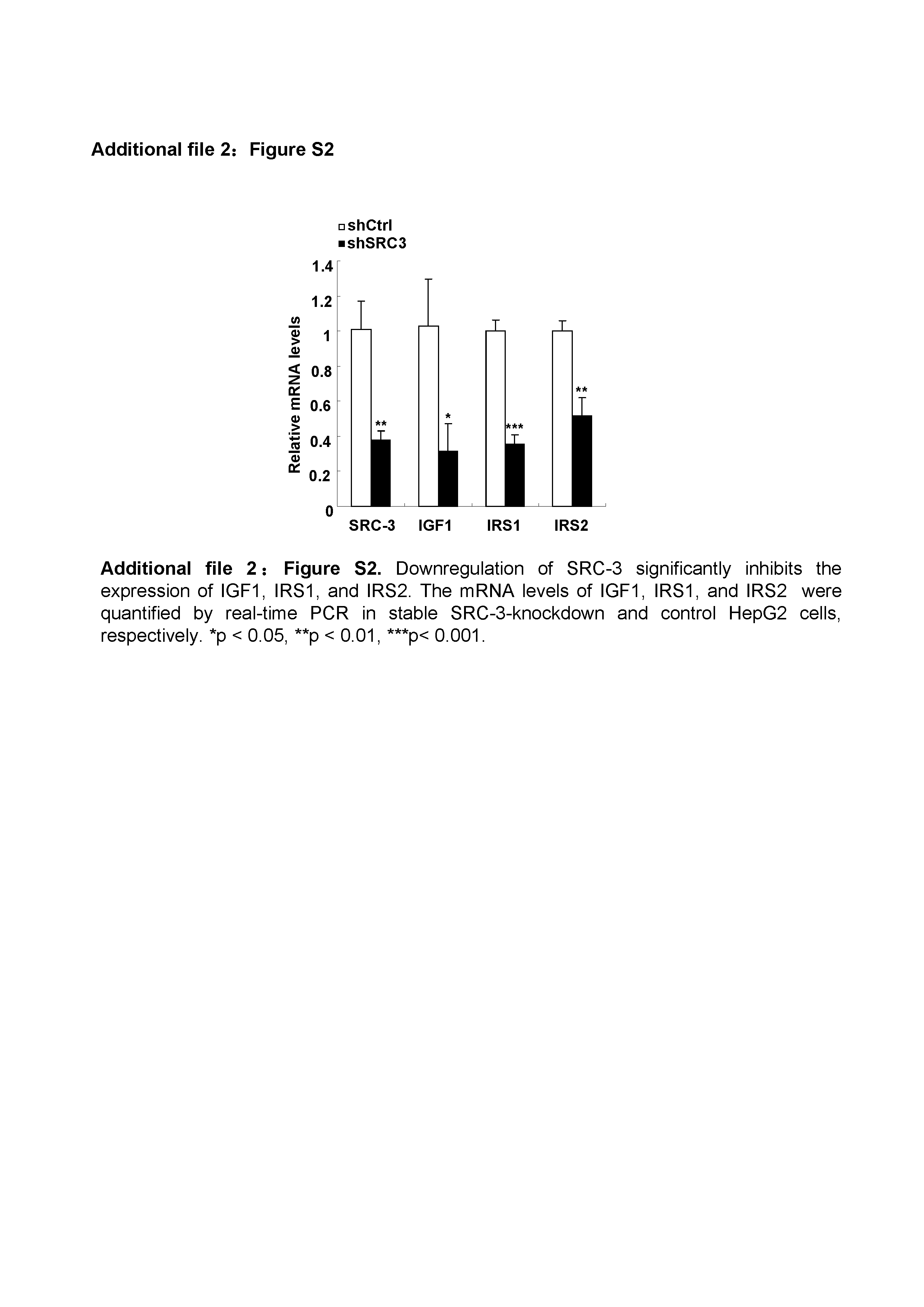

Supplement: Supplementary file 2 — Additional file 2: Figure S2. Downregulation of SRC-3 significantly inhibits the expression of IGF1, IRS1, and IRS2. The mRNA levels of IGF1, IRS1, and IRS2 were quantified by real-time PCR in stable SRC-3-knockdown and control HepG2 cells, respectively. *p < 0.05, **p < 0.01, ***p < 0.001. [file 13578_2019_328_MOESM2_ESM.tiff]
